# Identification of CDK1, PBK, and CHEK1 as an Oncogenic Signature in Glioblastoma: A Bioinformatics Approach to Repurpose Dapagliflozin as a Therapeutic Agent

**DOI:** 10.3390/ijms242216396

**Published:** 2023-11-16

**Authors:** Harold A. Chinyama, Li Wei, Ntlotlang Mokgautsi, Bashir Lawal, Alexander T. H. Wu, Hsu-Shan Huang

**Affiliations:** 1 Graduate Institute of Biomedical Informatics, College of Medical Science and Technology, Taipei Medical University, Taipei 11031, Taiwan; m610111011@tmu.edu.tw; 2 Department of Neurosurgery, Wan Fang Hospital, Taipei Medical University, No.111, Sec. 3, Xinglong Rd., Taipei 11696, Taiwan; 109058@w.tmu.edu.tw; 3 Taipei Neuroscience Institute, Taipei Medical University, Taipei 11031, Taiwan; 4 Graduate Institute of Injury Prevention and Control, College of Public Health, Taipei Medical University, Taipei 11031, Taiwan; 5 PhD Program for Cancer Molecular Biology and Drug Discovery, College of Medical Science and Technology, Taipei Medical University and Academia Sinica, Taipei 11031, Taiwan; d621108006@tmu.edu.tw; 6 Graduate Institute for Cancer Biology and Drug Discovery, College of Medical Science and Technology, Taipei Medical University, Taipei 11031, Taiwan; 7 Department of Pathology, University of Pittsburgh, Pittsburgh, PA 15232, USA; bal140@pitt.edu; 8 PhD Program of Translational Medicine, College of Medical Science and Technology, Taipei Medical University, Taipei 11031, Taiwan; 9 Clinical Research Center, Taipei Medical University Hospital, Taipei Medical University, Taipei 11031, Taiwan; 10 Graduate Institute of Medical Sciences, National Defense Medical Center, Taipei 11490, Taiwan; 11 School of Pharmacy, National Defense Medical Center, Taipei 11490, Taiwan; 12 PhD Program in Drug Discovery and Development Industry, College of Pharmacy, Taipei Medical University, Taipei 11031, Taiwan

**Keywords:** glioblastoma multiforme, cyclin-dependent kinase 1, PDZ binding kinase, checkpoint kinase 1, cell cycle, drug repurposing, Temozolomide, Dapagliflozin, Abemaciclib, molecular docking

## Abstract

Glioblastoma multiforme (GBM) is the most aggressive and lethal primary brain tumor whose median survival is less than 15 months. The current treatment regimen comprising surgical resectioning, chemotherapy with Temozolomide (TMZ), and adjuvant radiotherapy does not achieve total patient cure. Stem cells’ presence and GBM tumor heterogeneity increase their resistance to TMZ, hence the poor overall survival of patients. A dysregulated cell cycle in glioblastoma enhances the rapid progression of GBM by evading senescence or apoptosis through an over-expression of cyclin-dependent kinases and other protein kinases that are the cell cycle’s main regulatory proteins. Herein, we identified and validated the biomarker and predictive properties of a chemoradio-resistant oncogenic signature in GBM comprising CDK1, PBK, and CHEK1 through our comprehensive in silico analysis. We found that CDK1/PBK/CHEK1 overexpression drives the cell cycle, subsequently promoting GBM tumor progression. In addition, our Kaplan–Meier survival estimates validated the poor patient survival associated with an overexpression of these genes in GBM. We used in silico molecular docking to analyze and validate our objective to repurpose Dapagliflozin against CDK1/PBK/CHEK1. Our results showed that Dapagliflozin forms putative conventional hydrogen bonds with CDK1, PBK, and CHEK1 and arrests the cell cycle with the lowest energies as Abemaciclib.

## 1. Introduction

Glioblastoma Multiforme (GBM) remains the most aggressive primary brain tumor, with a poor response to aggressive management comprising surgical resection, chemotherapy, and radiotherapy with Temozolomide [1,2,3,4,5]. Despite this aggressive management approach, tumor progression and recurrence are almost inevitable in over 75% of the treated patients [6,7,8]. Tumor heterogeneity and stem cells’ presence enhance the therapy resistance and the consequential poor overall survival of GBM patients, averaging 15 months [1,2,9,10,11,12]. In addition, therapy combination only achieves a 10% five-year survival rate, prompting an urgent need to identify and validate theragnostic markers that will necessitate effective treatment strategies to improve glioblastoma patients’ quality of life and survival rate [7,13,14].

An aberrant cell cycle is a crucial cancer characteristic in which malignant cells develop and progress through uncontrolled cell proliferation and programmed cell death evasion [15,16,17,18]. Cyclin-dependent kinases (CDKs) and protein kinases are the main regulatory proteins for the cell cycle whose expression reflects the proliferative state of the tumor and correlates negatively with patient survival [9,19,20,21]. An in-depth understanding of the mechanisms with which these protein kinases promote cancer development and progression through the cell cycle is vital in precision oncology. We envisage that targeting these protein kinases, specifically CDK1/PBK/CHEK1 oncogenic signature, which we identified through in silico analysis, promotes the Gap 2 to mitosis (G2-M) phase cell cycle arrest, apoptosis, and therapy-induced senescence, hence inhibiting GBM progression [20,22,23,24]. Moreover, this will enhance therapy-induced and host immune system-mediated GBM senolyses before their eventual senescence-associated secretory phenotype (SASP) [22,23,24].

CDK1 is a central regulator of cell cycle progression, specifically the transition from the G2 to the M phases [19,25,26]. The activation of CDK1 by cyclins A and B1 (CCNB1) promotes M phase entry from the G2 phase [26,27]. CDK1 remains active until CCNB is degraded in the late M phase in readiness for cytokinesis [26,27]. However, CDK1′s dysregulated activation and high expression confer resistance to chemo and radiation therapy and correlate positively with poor patient prognosis in breast, liver, colorectal, prostate, and other cancers [18,20,25,28]. The CCNB1/CDK1 complex phosphorylates PDZ binding Kinase (PBK) in the late M phase before the complex is neutralized [29,30,31,32,33]. Activated PBK is required for the proper separation of chromosomes, completion of mitosis, and cytokinesis [29,30,31,32,33]. Its over-expression in cancer promotes cancer progression by enabling the successful completion of cytokinesis and tumor proliferation. PBK is over-expressed in GBM stem cells compared to normal neural stem cells, and its targeting suppresses proliferation and promotes apoptosis [1]. DNA damage enhances Checkpoint Kinase 1 (CHK1/CHEK1) activation and the subsequent arrest of cell cycle transition from the G2 to the M phases by directly phosphorylating WEE1 G2 Checkpoint Kinase (WEE1) [20,27,34]. WEE1 phosphorylates and inhibits CDK1′s activity in the G2 phase, blocking the cycle’s progression to the M phase [20,27]. This blockage results in DNA damage repair, cellular quiescence, senescence, or apoptosis should the DNA damage repair fail [20,27]. CHK1 enhances DNA repair in tumors, especially those with a defective p53, enhancing cancer cell survival and proliferation [27]. CHEK1 inhibition results in premature progression into the M phase of cells with an accumulation of DNA damages leading to M phase arrest and apoptosis [27]. There is, therefore, crosstalk amongst our chemo- and radio-resistant oncogenic signature comprising CDK1, PBK, and CHEK1 in the G2/M phase of the cell cycle.

The crosstalk among CDK1, PBK, and CHEK1 in the G2/M phase of the cell cycle is a potential therapeutic target and prognostic marker in GBM. Studies have shown that the selective inhibition of CDK1, PBK, and CHEK1 arrests tumor progression in sarcomas, GBM, prostate, endometrial, and breast cancers by blocking the G2-M phase transition and promoting apoptosis [1,9,35,36,37,38,39,40,41]. However, the simultaneous inhibition of the chemo radio-resistant CDK1/PBK/CHEK1 oncogenic signature in glioblastoma has never been explored despite the existing cell cycle crosstalk amongst these genes. This study discusses our assessment of CDK1/PBK/CHEK1′s potential as theragnostic and prognostic markers in glioblastoma. We further analyzed the interaction of Dapagliflozin, an FDA-approved type 2 diabetic drug, and our identified oncogenic signature in silico to ascertain the anti-tumor effect of this drug compared to Temozolomide.

## 2. Results

### 2.1. DEG Identification and Analysis in GBM

**Figure 1:**
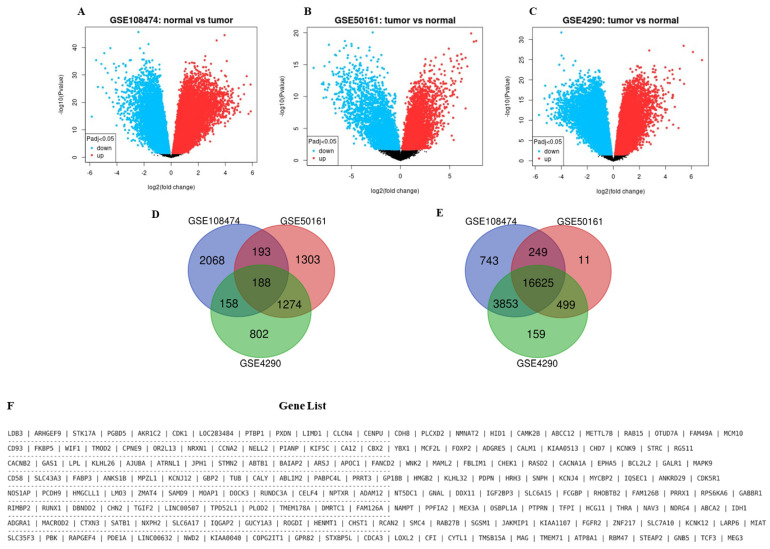
Differentially expressed genes (DEGs) from GSE108474, GSE50161, and GSE4290 comprise gene expression profiles of GBM. (**A**–**C**) Depict the DEGs from GSE108474, GSE50161, and GSE4290 GBM samples with a *p*-value set at <0.05. The red and blue dots represent up and downregulated genes, respectively. (**D**) A Venn diagram with 188 overlapping overexpressed genes while (**E**) comprises 16,625 overlapping-downregulated genes analyzed in BEG. (**F**) is a list of the 188 overlapping overexpressed genes.

We generated volcano plots and a list of DEGs on GEO2R by analyzing GSE108474, GSE50161, and GSE4290 comprising gene expression profiles of GBM patients’ samples. The samples were pre-classified as tumor and normal with the significant level cut-off value set at <0.05 (adj *p*-value), -10 log, and -2-fold change. We further analyzed the resultant DEGs from GSE108474, GSE50161, and GSE4290 in BEG to identify overlapping genes. In total, 188 upregulated-overlapping genes and 16,625 downregulated overlapping genes were obtained from the analysis, as shown in the Venn diagrams labeled Figure 1D,E below. Only the overlapping overexpressed genes’ list was sorted and used in Python to generate the gene list in Figure 1F below.

### 2.2. PPI Network Construction and Associated Functional Enrichment

**Figure 2:**
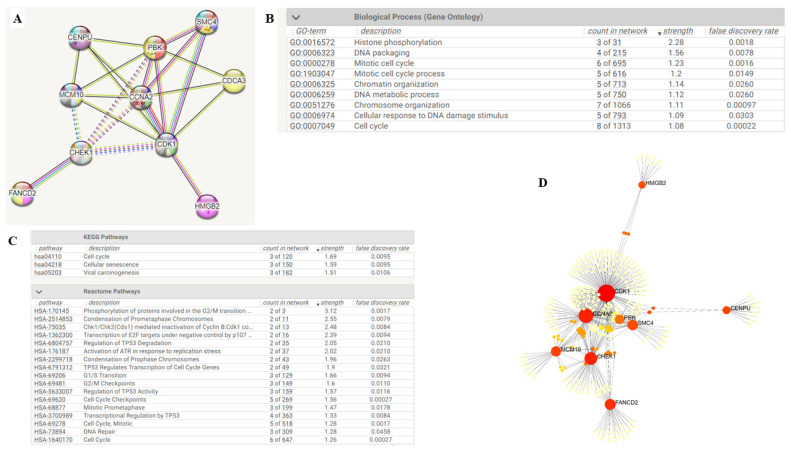
Clustered PPI networks comprising CDK1/PBK/CHEK1 oncogenes in GBM and their functional enrichments. (**A**) A PPI cluster with a minimum interaction score significance > 0.700, 10 nodes, and 22 edges with an average local clustering coefficient of 0.82 and a PPI enrichment *p*-value of 2.11 × 10^−15^. The PPI network is from co-expression, text mining, databases, experiments, co-occurrence, neighborhood, and gene fusion. (**B**,**C**) show functional enrichments under the Gene Ontology (GO) biological processes, Kyoto Encyclopedia of Genes and Genomes (KEGG), and Reactome pathways that were available upon an analysis of the PPI network in the STRING database. (**D**) A signaling network cluster of KEGG enrichment analysis revealing CDK1/PBK/CHEK1 co-expression in the cell cycle analyzed in Network Analyst.

A PPI network analysis on the STRING database revealed a high-confidence network comprising CDK1, PBK, and CHEK1, with a minimum interaction score significance > 0.700. The network had 10 nodes and 22 edges within its cluster, with an average local clustering coefficient of 0.82 and a PPI enrichment *p*-value of 2.11 × 10^−15^. We constructed the PPI network shown in Figure 2A based on protein co-expression, text mining, databases, experiments, co-occurrence, neighborhood, and gene fusion. We also extracted the functional enrichments under the Gene Ontology (GO) biological processes, Kyoto Encyclopedia of Genes and Genomes (KEGG), and Reactome pathways available upon analysis of the PPI network, as shown in Figure 2B,C below. The FDR for all pathways was <0.05, with most being <0.01, signifying their significance. We further analyzed the PPI network on Network Analyst for better visualization and functional enrichments.

### 2.3. CDK1/PBK/CHEK1 Are Overexpressed and Highly Correlated in GBM

**Figure 3:**
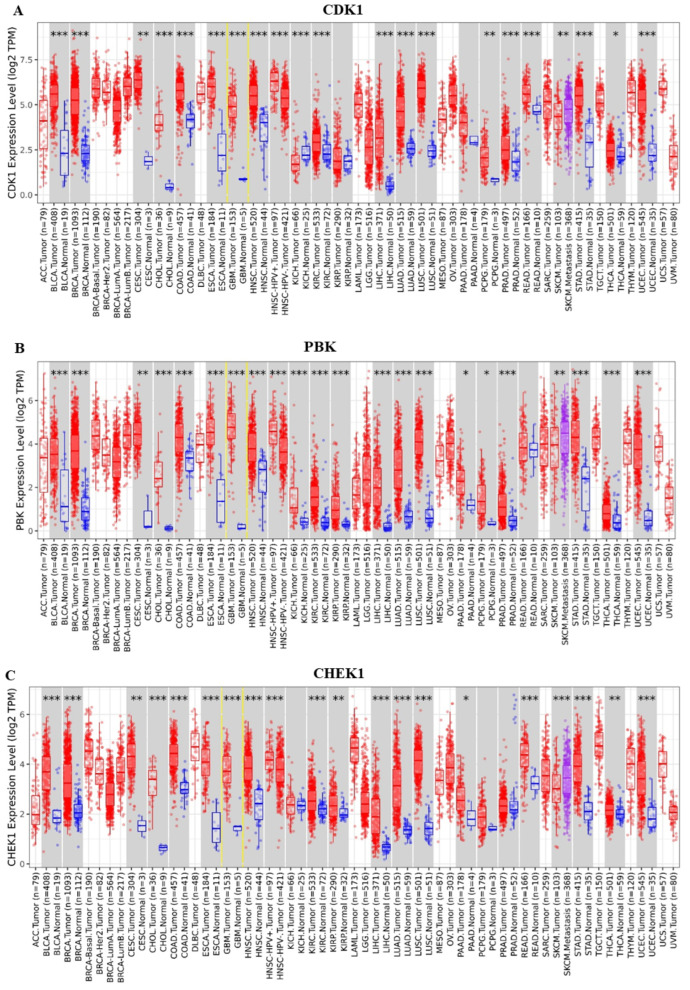

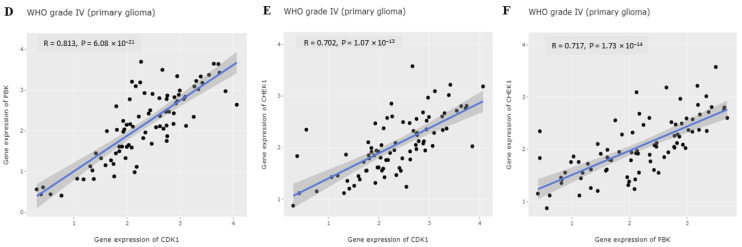
CDK1/PBK/CHEK1 expression and correlation profiling in GBM. (**A**–**C**) CDK1/PBK/CHEK1 is overexpressed in GBM and other cancer types from the TCGA and was analyzed on TIMER2.0. Their mRNA expression levels are expressed as TPM and were normalized and transformed using log transformation with a *p*-value significance code of 0 ≤ *** < 0.001 ≤ ** < 0.01 ≤ * < 0.05 ≤ . < 0.1. (**D**–**F**) represent the positive correlation of CDK1/PBK/CHEK1 in WHO grade IV primary glioma (GBM), with R ranging from 0.702 to 0.813 and a *p*-value < 0.05 affirming the statistical significance of the association of the CDK1/PBK/CHEK1 oncogenic signature in GBM.

We used TIMER2.0 and CGGA for a comprehensive and systematic CDK1/PBK/CHEK1 oncogenic signature analysis for their differential expression in pan cancers and their correlation in GBM. These genes were highly expressed in 153 GBM samples compared to five adjacent tissues, as evaluated by the Wilcoxon test on TIMER2.0 and expressed as transcripts per million (TPM) with a *p*-value significant code of 0 ≤ *** < 0.001. The gene expression data were transformed and normalized through log transformation. A correlation analysis on CGGA predicted a positive correlation of the genes in GBM with Pearson correlation coefficients (R) of 0.702, 0.717, and 0.813 among them and a *p*-value < 0.05, signifying a statistically significant relationship. This strong positive correlation could enhance and promote tumor aggressiveness, growth, metastasis, survival, and drug resistance in GBM whose understanding could potentially help develop targeted therapy and serve as prognostic markers. Figure 3 below shows the differential expression and correlation relationship obtained from TIMER2.0 and CGGA.

### 2.4. CDK1/PBK/CHEK1 Overexpression Is Associated with the Late-Stage GBM

**Figure 4:**
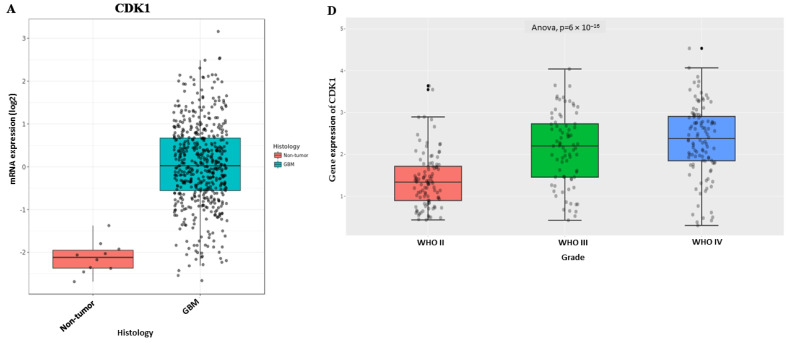

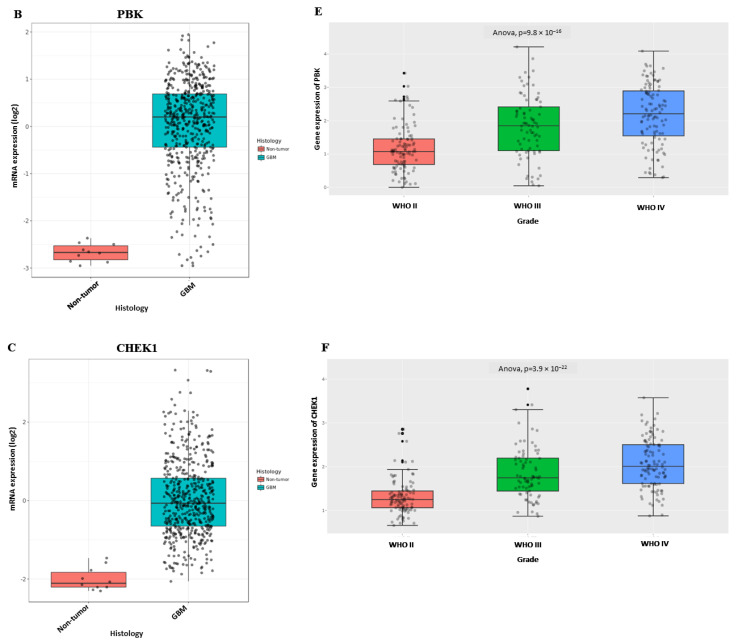
CDK1/PBK/CHEK1 overexpression is associated with the late-stage GBM. (**A**–**C**) The CDK1/PBK/CHEK1 level overexpression was elevated in the GBM samples normalized and transformed using log2 transformation with a *p*-value < 0.05 considered statistically significant on the GlioVis tool. (**D**–**F**) Boxplots generated from the CGGA analysis reflecting CDK1/PBK/CHEK1 gene overexpression in three WHO glioma grades II, III, and IV with a *p*-value < 0.05.

We further validated the differential expression of CDK1, PBK, and CHEK1 in GBM using the CGGA database and GlioVis (http://gliovis.bioinfo.cnio.es/), an online application for the expression of genes in glioblastoma. The analytical results in Figure 4A–C below from GlioVis show an overexpression of CDK1, PBK, and CHEK1 in GBM than in nontumor. We used log2 transformation to normalize, stabilize, and interpret the differential expression of the mRNAs in nontumor and GBM on GlioVis. Using the analysis of variance (ANOVA) and the *p*-value < 0.05, we validated that CDK1, PBK, and CHEK1 are all overexpressed in WHO glioma grades II-IV as illustrated in Figure 4D–F below, obtained from the analysis of the genes on the CGGA.

### 2.5. A High CDK1/PBK/CHEK1 Expression Promotes Immune Evasion and Tumor Aggressiveness in GBM

**Figure 5:**
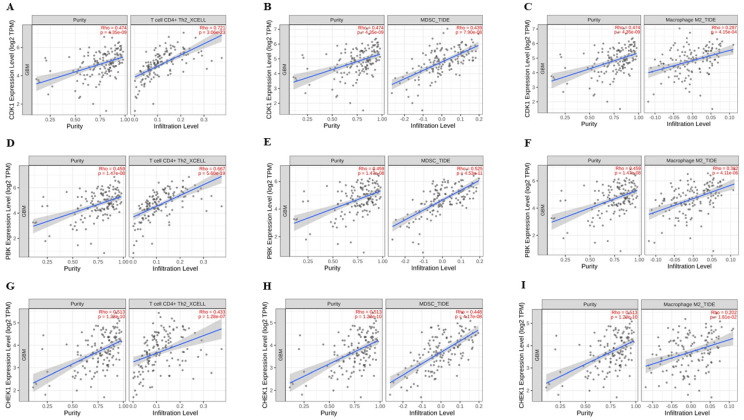
CDK1/PBK/CHEK1 overexpression promotes tumor aggressiveness and immunosuppression in GBM. (**A**–**C**) depict a moderate positive correlation of CDK1 expression levels with tumor purity (Rho = 0.474) and a strong, moderate, and weak positive correlation with T Cells CD4+ Th2 (Rho = 0.721), MDSCs (Rho = 0.439), and TAM-M2 (Rho = 0.297) infiltration levels in GBM, analyzed on TIMER2.0. (**D**–**F**) show a moderate positive correlation of PBK expression level with GBM tumor purity (Rho = 0.459), as well as a strong, moderate, and weak positive correlation with T Cells CD4+ Th2 (Rho = 0.667), MDSCs (Rho = 0.525), and TAM-M2 (Rho = 0.382) infiltration levels. (**G**–**I**) show a moderate positive correlation of CHEK1 expression levels with tumor purity (Rho = 0.513), T Cells CD4+ Th2 (Rho = 0.433), and MDSCs (Rho = 0.448), and a weak positive correlation with TAM-M2 (Rho = 0.202) infiltration levels in GBM. CDK1/PBK/CHEK1 mRNA expression levels are expressed as TPM and were normalized and transformed using log2 transformation with a *p*-value < 0.05.

All the results discussed below had a *p*-value < 0.05. There was a moderate positive correlation between tumor purity and CDK1/PBK/CHEK1 with a Rho of 0.474, 0.459, and 0.513. These results estimate that CDK1/PBK/CHEK1 overexpression promotes tumor aggressiveness by promoting its growth and GBM tumor cell population within the tumor microenvironment [42]. Our analysis also estimated a strong positive correlation between CDK1 and PBK expression levels and T Cells CD4+ Th2 infiltration levels with a Rho of 0.721 and 0.667. The moderate positive correlation between the CHEK1 expression level and the T Cells CD4+ Th2 infiltration level had an Rho of 0.433. In addition, a moderate positive correlation of the CDK1, PBK, and CHEK1 expression levels with the MDSC infiltration levels had a Rho of 0.439, 0.525, and 0.448. However, our analytical results had a weak positive correlation of our oncogenic signature with TAM-M2 with a Rho of 0.297, 0.382, and 0.202. These putative positive correlations show that a high CDK1/PBK/CHEK1 expression promotes tumor growth, aggressiveness, and immune evasion through the increased recruitment of T Cells CD4+ Th2, MDSCs, and TAM-M2, all of which have pro-tumor effects. These immune cells promote tumor angiogenesis and suppress other anti-tumor immune responses resulting in metastasis and poor patient survival [42,43,44,45,46]. The scatter plots in Figure 5 below show these analytical results.

### 2.6. CDK1/PBK/CHEK1 Overexpression in GBM Is Associated with Poor Patient Survival

We predicted and validated the prognostic value of CDK1, PBK, and CHEK1 in the GlioVis database and constructed radiomics signatures whose determined maximally selected rank statistics cut-off values were 1.55, 0.81, and 1.42. These results mean patients whose Radscores were low but had a high expression of CDK1, PBK, and CHEK1 had a poor prognosis, signifying the significance of these oncogenes in the cell cycle and their potential to be prognostic biomarkers in GBM.

**Figure 6:**
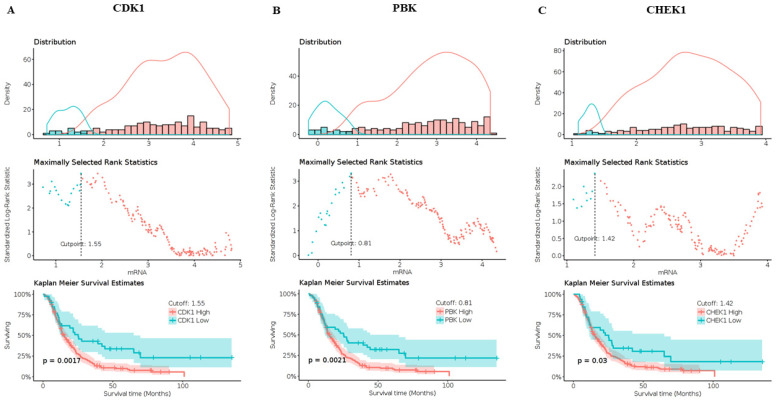
CDK1/PBK/CHEK1 overexpression in GBM is associated with poor patient survival. (**A**–**C**) show a poor prognosis of the highly expressed CDK1, PBK, and CHEK1 oncogenes in GBM with low cut-off values of 1.55, 0.81, and 1.42. KM survival graphs predict a shorter overall survival for the highly expressed oncogenes with a *p*-value < 0.05. The high expression of the oncogenes is indicated in red with poor overall survival compared to low expressions (in blue) of the same in GBM.

We further validated the prognostic significance of CDK1, PBK, and CHEK1 in GBM using the KM survival analysis plotted in GlioVis. Prolonged overall survival in GBM patients had an inversely proportional relationship with the high expression of CDK1, PBK, and CHEK1 at a *p*-value < 0.05 on the KM graphs, validating the potential prognostic value of the three genes in GBM. The graphs in Figure 6 below show the potential prognostic value of CDK1/PBK/CHEK1 oncogenic signature in GBM.

### 2.7. Molecular Docking Analysis Confirms Dapagliflozin as a Potential Therapeutic Agent for Targeting CDK1/PBK/CHEK1 in GBM

The molecular docking results confirmed the potential of Dapagliflozin in inducing apoptosis by arresting the cell cycle by targeting CDK1, PBK, and CHEK1 in glioblastoma. Dapagliflozin showed lower putative binding energies with CDK1 [−8.4 kcal/mol of Gibbs free energy (ΔG)], PBK [ΔG = −7.2 kcal/mol], and CHEK1 [ΔG = −8.3 kcal/mol] oncogenes through our docking analysis performed using AutoDock Vina software in the command prompt. We compared Dapagliflozin’s binding affinity to CDK1 to Abemaciclib’s binding affinity to CDK1, obtained using the same analytical method with CDK1 having a ΔG of −8.9 kcal/mol, suggesting a similar better effect of Dapagliflozin in targeting these oncogenes but with a shorter binding distance. However, we found no FDA-approved standard inhibitor for PBK and CHEK1 to compare as references for our molecular docking results for these oncogenes. Moreover, analytical results in BIOVIA Discovery Studio revealed that Dapagliflozin formed conventional hydrogen (H) bonds with CDK1 on GLN132 (1.94 Å, 2.72 Å), Alkyl bond on ALA145, Pi-Alkyl bonds (ALA132, LEU135, PHE82, VAL18), and Pi-Sigma on PHE82.

**Figure 7:**
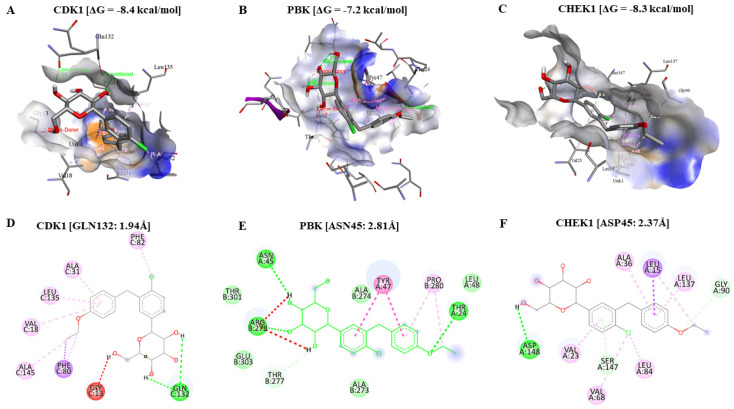
Putative binding interactions of Dapagliflozin with CDK1, PBK, and CHEK1 in 3D and 2D structures. (**A**,**D**) Dapagliflozin binding on CDK1 with lower putative binding energy [ΔG = −8.4 kcal/mol] and a short binding distance (GLN132:1.94 Å) stabilized by a H bond in green color. (**B**,**E**) represent lower putative binding energy [ΔG = −7.2 kcal/mol] and short binding distance (ASN45:2.81 Å) of Dapagliflozin on PBK. (**C**,**F**) confirm that Dapagliflozin has higher binding affinity, evidenced by the lower Gibbs free energy [ΔG = −8.3 kcal/mol] on CHEK1 stabilized by H bond (ASP148:2.37 Å).

**Figure 8:**
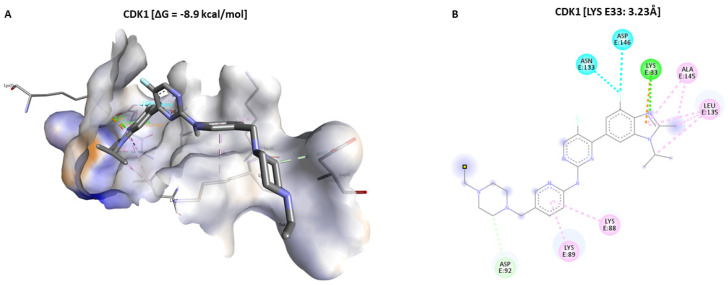
Putative binding of Abemaciclib on CDK1 in 3D and 2D structures. (**A**,**B**) show a slightly better putative binding of Abemaciclib on CDK1 [ΔG = −8.9 kcal/mol] but has a longer binding distance (LYS33:3.23 Å) stabilized by a H bond in green color compared to Dapagliflozin.

PBK had three H bonds with Dapagliflozin (ASN45:2.81Å, ARG278:2.96Å, and THR24:3.07Å), Alkyl and Pi-Alkyl bonds on PRO280, Carbon Hydrogen Bond on THR277, and Pi-Pi-T shaped and Pi-Pi Stacked on TYR47. CHEK1 formed a H bond with Dapagliflozin on ASP148 (2.37Å), Alkyl (VAL68, LEU15 and LEU84), Pi-Alkyl (LEU137, ALA36, VAL23), Carbon Hydrogen Bond on GLY90, Pi-Sigma on LEU15, and Pi-Donor Hydrogen bond on SER147. Figure 7 and Figure 8 below are a 3D and 2D graphical presentation of these interactions as viewed in BIOVIA Discovery Studio. The details of all the interactions between Dapagliflozin and CDK1, PBK, and CHEK1 are summarized in Table 1 below.

## 3. Discussion

There is still no cure for glioblastoma, whose aggressiveness results in poor overall patient survival, with a median survival of less than 15 months [5]. Temozolomide resistance contributes to poor patient survival in GBM despite TMZ being the only approved chemotherapeutic drug for GBM [47,48,49]. In addition, GBM tumor heterogeneity and stem cell presence complicate total patient cure even after surgical resection, chemotherapy, and adjuvant radiation therapy [50]. It is imperative to continue exploring other treatment options that will sensitize tumors and improve the current survival rate of GBM patients.

Accumulating studies have shown that CDKs and other protein kinases are potential therapeutic targets in GBM and other cancers due to the vital roles these kinases play in driving the cell cycle and eventual tumor progression [1,20,27,32,38,51,52]. We identified a chemoradio-resistant oncogenic signature in GBM comprising CDK1, PBK, and CHEK1 through our comprehensive in silico analysis. We explored various databases to comprehensively identify and analyze this oncogenic signature in GBM and the role it plays in promoting tumor progression and overall patient survival. In this study, we successfully showed that the overexpression of these genes promotes tumor progression by aiding its proliferation, evading senescence and apoptosis through the aberrant completion of the cell cycle, especially the G2/M phase transition. In addition, we showed that CDK1/PBK/CHEK1 overexpression promotes GBM tumor aggressiveness, immunosuppression, and progression by recruiting the tumor-promoting immune cells such as the T Cells CD4+ Th2 subtype, MDSCs, and the TAM-M2 subtype [42,46,53,54]. These pro-tumor immune cells enhance tumor initiation and angiogenesis and suppress other anti-tumor immune responses, increasing tumor cell population within the tumor microenvironment, metastasis, and eventual poor patient survival [43,44,45,55,56]. Furthermore, we revealed that CDK1/PBK/CHEK1′s overexpression is associated with poor patient survival in GBM as outlined above in the KM survival estimates, thus underlining the biomarker and prognostic properties of the CDK1/PBK/CHEK1 oncogenic signature in GBM.

Despite advancements in unraveling potential therapeutic targets in GBM, several attempts to develop other drugs and treatment modalities for this aggressive primary brain tumor have not been successful. The blood–brain barrier (BBB) is the main reason for this failure due to the presence of efflux pumps that actively pump out cancer drugs that cross the BBB [57,58]. Moreover, some of the developed drugs have been toxic to normal cells, outweighing the benefits that they might have in killing cancer cells [51,59,60]. However, studies have shown that Sodium Glucose-linked cotransporters 2 (SGLT2) inhibitors such as Dapagliflozin cross the BBB as the brain has SGLT2 receptors [61,62]. In addition, Dapagliflozin is less toxic and promotes cardiovascular, renal, and neurovascular protection [62,63]. It has since been successfully repurposed and approved for treating chronic kidney diseases [63,64]. Studies have shown the potential anticancer effects that SGLT2 inhibitors have in addition to their primary correlation with glucose transporters [65,66,67,68]. In addition, studies have confirmed the inhibitory effect of SGLT2 inhibitors such as Canagliflozin on CDK1 and the subsequent cell cycle arrest at the G2/M in hepatocellular carcinoma [67]. Furthermore, Dapagliflozin has previously shown inhibitory effects on different cancer types’ cell adhesion by acting directly on the ADAM10 gene [67]. In this study, we have successfully shown and validated through in silico molecular docking and analyses that Dapagliflozin binds to CDK1, PBK, and CHEK1 and arrests the cell cycle progression from the G2/M phase with equally the lowest energies as Abemaciclib. These interactions are well stabilized by conventional hydrogen bonds. The simultaneous inhibition of CDK1/PBK/CHEK1 will promote the cell cycle arrest, senescence, and apoptosis of GBM, suppressing tumor progression. Our results elucidate the anticancer properties of Dapagliflozin, an already approved drug with a well-documented safety profile, as a potential drug for glioblastoma treatment [67,69]. It has previously been reported that a combination of cancer therapy comprising Dapagliflozin or other SGLT2 inhibitors enhances patient tolerance to standard cancer treatments and improves the drugs’ effectiveness [67,68,69,70]. Preclinical experiments are ongoing in our laboratory to validate the efficacy and therapeutic effect of Dapagliflozin on CDK1/PBK/CHEK1 and evaluate the viability of a combination therapy with standard treatments to improve the current chemo-radiotherapy resistance and toxicity status in GBM [49,59,67,68,69,70,71,72].

## 4. Materials and Methods

### 4.1. Gene Expression Dataset Retrieval

GSE108474, GSE50161, and GSE4290, comprising gene expression profiles of GBM patients’ samples, were downloaded from Gene Expression Omnibus (GEO) database (https://www.ncbi.nlm.nih.gov/gds/ accessed on 14 February 2023), a functional genomics data repository available for public use [73,74]. The samples were then sorted into tumor and normal before being analyzed for differentially expressed genes (DEGs) on an interactive web tool, GEO2R (https://www.ncbi.nlm.nih.gov/geo/geo2r/ accessed on 14 February 2023) [75]. The False discovery rate (FDR) was controlled by adjusting the *p*-values to enhance the sensitivity by opting for the Benjamini and Hochberg method, with the fold-change threshold and the significance level cut-off set at 1.5 and 0.05, respectively. These DEGs were sorted and analyzed on Bioinformatics & Evolutionary Genomics (BEG), an online tool available at http://bioinformatics.psb.ugent.be/webtools/Venn/ (accessed on 14 February 2023) to identify overlapping genes and visualize them in Venn diagrams drawn using this tool.

### 4.2. Protein–Protein Interaction (PPI) Network and Functional Enrichment Analysis

We explored the STRING database, version 11.5, accessible at https://string-db.org/ (accessed on 30 March 2023), to predict the interactions of the overlapping overexpressed genes [76,77,78]. We clustered the PPI networks and extracted statistically significant (*p*-value < 0.05) functional enrichments under the Gene Ontology (GO) biological processes, Reactome, and Kyoto Encyclopedia of Genes and Genomes (KEGG) pathways. We identified our oncogenic signature comprising CDK1, PBK, and CHEK1 from the PPI network cluster. We further analyzed the PPI network on Network Analyst, available at https://www.networkanalyst.ca/NetworkAnalyst/ (accessed on 1 April 2023), which provides comprehensive profiling and visual analytics of gene expression and networks, respectively [79,80,81].

### 4.3. Validation of CDK1/PBK/CHEK1 Expression and Correlation in GBM

We validated the identified gene signature on the Tumor Immune Estimation Resource (TIMER2.0) available at http://timer.cistrome.org/ (accessed on 16 March 2023) [82]. We aimed to explore the individual differential expression of these genes between tumor and normal samples based on their availability in The Cancer Genome Atlas (TCGA) and in pan-cancer with a primary focus on GBM. We further validated CDK1/PBK/CHEK1 and explored their correlation on the Chinese Glioma Genome Atlas (CGGA) accessible at http://www.cgga.org.cn/index.jsp (accessed on 16 March 2023) [83]. Only those DEGs with a *p*-value < 0.05 and a positive Pearson correlation coefficient were considered significant.

### 4.4. Correlation between CDK1/PBK/CHEK1 Overexpression and Immune Cell Infiltration Levels

We analyzed the correlation between the overexpression of CDK1/PBK/CHEK1 and the immune cells namely T cell CD4+ Th2, myeloid-derived suppressor cells (MDSC), and tumor-associated macrophages M2 subtype (TAM-M2) in GBM using TIMER2.0. TIMER2.0 has a comprehensive set of algorithms used for estimating and analyzing the immune infiltration levels of pan cancers available in TCGA [82]. We selected the tumor purity adjustment option to use the partial Spearman’s correlation (Rho) to control confounding variables in our analysis since tumor purity is the major confounder in this association analysis and is often negatively associated with most immune cell types [82]. We focused on the analytical results from the tumor immune dysfunction and exclusion (TIDE) and xCell algorithms of TIMER2.0 due to the statistical significance of the correlation between CDK1/PBK/CHEK1 and the immune cells with *p*-value < 0.05 [84,85]. These algorithms use gene expression profiles and gene set enrichment analysis data to compute tumor purity, stromal cell presence in tumor microenvironment, T-cell dysfunction, and T-cell exclusion in tumors and give a comprehensive estimate.

### 4.5. Assessment of CDK1, PBK, and CHEK1 as Prognostic Biomarkers in GBM

We used GlioVis (http://gliovis.bioinfo.cnio.es/ accessed on 16 March 2023) to assess the prognostic value of CDK1, PBK, and CHEK1 in glioblastoma, and we selected the CGGA dataset utilizing the RNA-Seq data for its estimation [86]. Furthermore, we opted for all subtypes, all genders, and all IDH statuses for our analysis. We estimated the Kaplan–Meier survival and analyzed the maximally selected rank statistics for the genes’ cut-off value on GlioVis with a *p*-value < 0.05.

### 4.6. Molecular Docking Analysis

We used the molecular docking analysis to assess the interactions and effects of Dapagliflozin (CID:9887712), which we aim to repurpose against CDK1, PBK, and CHEK1 in GBM [87,88]. In addition, we analyzed CDK1 interactions with Abemaciclib (CID:46220502), an FDA-approved standard inhibitor against CDKs, as a reference during our results analysis due to its documented inhibitory activity against CDK1 [89]. However, we found no FDA-approved standard inhibitor for PBK and CHEK1 to compare our molecular docking results. We downloaded 3D structures of the ligands, Dapagliflozin and Abemaciclib, in SDF format on PubChem (https://pubchem.ncbi.nlm.nih.gov/ accessed on 2 April 2023) and converted them to PDB format in Open Babel GUI version 3.1.1. The CDK1 (PDB:4YC6), PBK (PDB:5J0A), and CHEK1 (PDB:2HOG) proteins’ 3D crystal structures were downloaded from RSCB protein data bank (RSCB PDB), accessed on https://www.rcsb.org/ (accessed on 2 April 2023) [90,91,92,93]. Further pre-processing of the ligands and proteins (receptors) and their subsequent conversion to PDBQT formats were performed in AutoDockTools-1.5.7 [94]. We used AutoDockVina commands in the command prompt to perform molecular docking for our receptors and the ligands, with the results analyzed in PyMOL version 2.5.4 and BIOVIA Discovery Studio 2021 Client [95,96,97].

## 5. Conclusions

In conclusion, through our comprehensive in silico analysis, we showed that overexpression and a strong positive correlation of the CDK1/PBK/CHEK1 oncogenic signature in GBM promote its aggressiveness, immuno-evasion, and tumor progression through an aberrant completion of the cell cycle and recruitment of the tumor-promoting immune cells such as the T Cells CD4+ Th2 subtype, MDSCs, and the TAM-M2 subtype. Furthermore, our KM survival estimates unraveled the poor patient survival associated with a high CDK1/PBK/CHEK1 expression in GBM. In light of this, we showed that this chemo-radio-resistant oncogenic signature is a potential prognostic biomarker and a potential target for Dapagliflozin. Our molecular docking analysis showed that Dapagliflozin binds to CDK1, PBK, and CHEK1 with equally the lowest Gibbs free energies like those of the FDA-approved standard inhibitor used. We will use the ongoing preclinical experiments in our laboratory to validate our findings and evaluate the viability of a combination therapy.

## Figures and Tables

**Table 1:** A summary of the docking results for Dapagliflozin on CDK1/PBK/CHEK1 oncogenic signature. The results comprise the binding energies, binding distances, the interacting amino acid on the receptor, and the type of bond for each ligand–receptor interaction.

Oncogenes and Binding Energy []	Interacting Amino Acids and Binding Distance ()	Interaction Type
CDK1 [ΔG = −8.4 kcal/mol]	GLN132 (1.94 Å, 2.72 Å)	Conventional Hydrogen Bond
	ALA145	Alkyl
	ALA132, LEU135, PHE82, VAL18	Pi–Alkyl
	PHE82	Pi–Sigma
PBK [ΔG = −7.2 kcal/mol]	ASN45 (2.81 Å), ARG278 (2.96 Å), THR24 (3.07 Å)	Conventional Hydrogen Bond
	PRO280	Alkyl and Pi–Alkyl bonds
	THR277	Carbon Hydrogen Bond
	TYR47	Pi-Pi-T shaped and Pi-Pi Stacked
CHEK1 [ΔG = −8.3 kcal/mol]	ASP148 (2.37 Å)	Conventional Hydrogen Bond
	VAL68, LEU15, LEU84	Alkyl
	LEU137, ALA36, VAL23	Pi–Alkyl
	GLY90	Carbon Hydrogen Bond
	LEU15	Pi–Sigma
	SER147	Pi–Donor Hydrogen bond

## Data Availability

All the generated and analyzed datasets of this study are available upon request.
